# The association of down-regulated toll-like receptor 4 expression with airflow limitation and emphysema in smokers

**DOI:** 10.1186/1465-9921-13-106

**Published:** 2012-11-21

**Authors:** Sei Won Lee, Dal Rae Kim, Tae Jung Kim, Jin Ho Paik, Jin-Haeng Chung, Sanghoon Jheon, Jin Won Huh, Jae-Ho Lee, Choon-Taek Lee

**Affiliations:** 1 Department of Pulmonology and Critical Care Medicine, Asan Medical Center, University of Ulsan College of Medicine, Seoul, Republic of Korea; 2 Division of Pulmonology and Critical Care Medicine, Department of Internal Medicine and Respiratory Center, Seoul National University Bundang Hospital, Seongnam, Republic of Korea; 3 Department of Radiology, Seoul National University Bundang Hospital, Seongnam, Republic of Korea; 4 Department of Pathology, Seoul National University Bundang Hospital, Seongnam, Republic of Korea; 5 Department of Thoracic Surgery, Seoul National University Bundang Hospital, Seongnam, Republic of Korea; 6 Department of Internal Medicine, Lung Institute, Seoul National University College of Medicine, Seoul, Republic of Korea

**Keywords:** Smoking, Emphysema, COPD, Toll-like receptor

## Abstract

**Background:**

An association between innate immunity including Toll-like receptors (TLRs) and COPD is reported recently; TLR4 deficiency in lung can cause emphysema in animals, which is not evident in humans. We analyzed the association of TLR4 expression, airflow limitation and emphysema in smokers.

**Methods:**

We enrolled patients of ≥40years old with smoking histories of ≥10 pack-years and who had undergone lung resection. We measured TLR4 expression in lung lysates. The severity of emphysema was evaluated on computed tomography. TLR4 expression was also evaluated immunohistochemically.

**Results:**

In total, 53 patients were enrolled. Forced expiratory volume in one second per forced vital capacity (FEV_1_/FVC) increased (*P*=0.03) and emphysema score decreased (*P*=0.01) as TLR4 expression increased. These were still significant, in multiple regression analysis including sex, age, tuberculosis history, smoking history and inhaled corticosteroid (ICS) usage. We also classified patients as high, intermediate, and low expressers according to TLR4 expression. Although no differences in age, gender, tuberculosis, or smoking history were observed among the groups, emphysema severity increased significantly (*P* = 0.02) and FEV_1_/FVC decreased significantly (*P* = 0.006) in TLR4 low expresser. The difference in TLR4 expression based on immunohistochemistry was most prominent in bronchial and alveolar epithelial cells.

**Conclusion:**

Down-regulated TLR4 expression in lung was associated with emphysema and airflow limitation in smokers.

## Background

Chronic obstructive pulmonary disease (COPD) is a major global health problem, with a prevalence of 5–25% in adults [1]. COPD is projected to be the fourth leading cause of death by 2020 [2,3]. Smoking is the most important cause of COPD, accounting for 90% of the cumulative risk [4]. Nevertheless, not all smokers will develop COPD. In fact, only a small portion (about 15%) will develop clinically relevant disease [5,6]. Compared with non-smokers, smokers have a steeper lung function decline. However, this is only true of susceptible smokers, as non-susceptible smokers can have the same lung function decline as never-smokers [7]. This implies that additional pathogenesis is involved in airway limitation and the development of emphysema.

Several candidate genes and biomarkers have been suggested to explain “additional pathogenesis” in smokers [8]. Candidate genes regulate proteases and antiproteases, modulate the metabolism of toxic substances in cigarette smoke, participate in mucocillary clearance, or influence inflammatory mediators [9], but the only well-established genetic risk factor is alpha-1-antitrypsin [10,11]. Microsatellite DNA instability has been proposed as a useful genetic screening marker for susceptible smokers [12]. However, unlike other diseases, COPD lacks established markers that can be applied to track disease progression [13].

Recently, some studies have reported an association between innate immunity and COPD. The main components of innate immunity are phagocytes of inflammatory cells, which discriminate between pathogens and self cells by utilizing signals from Toll-like receptors (TLRs). TLRs may be important in COPD because they participate in defense against viral and bacterial infections, and infections in the airway worsen the disease process in the lungs of patients with COPD [14]. Among the TLRs, TLR4 appears to play a pivotal role in lung homeostasis by contributing to the defense of endothelial cells against oxidants [15,16]. Moreover, TLR4 deficiency in the lung leads to spontaneous emphysema in animals, which is associated with an oxidant/antioxidant imbalance [17]. Thus, difference in TLR4 expression has been associated with COPD development in some animals, but this is not evident in humans.

In the present study, we hypothesized that TLR4 expression differs among people and that this can be associated with the emphysema or airflow limitation. To test this hypothesis, we analyzed the association between emphysema severity, TLR4 expression in lung tissue and emphysema.

## Material and methods

### Study patients

We recruited all patients with smoking history ≥10 pack-years, who were ≥40 years old, and whose lung had been resected for lung cancer from 2008 to 2010 at Seoul National University Bundang Hospital. We enrolled the patients whose normal lung tissues were available from a certified tissue bank. The lung tissues were dissected by pathologists to discriminate normal tissue from cancer and then preserved at −70°C until the experiment. The tissue was taken at peripheral area, at least 3cm apart from the cancer. This study was reviewed and approved by the Institutional Review Board of Seoul National University Bundang Hospital (B1008/110-006).

### Western blot assay

TLR4 expression was assessed by Western blot analysis. Normal lung tissues were homogenized (IKA T10 Basic; Younjin Corp., Gunpo, Korea) in lysis buffer (cell signaling, Denver, MA, USA) and 30ug protein was used for each sample. Western blot analysis was performed with anti-TLR4 antibody (H-80; Santa Cruz Biotechnology, Santa Cruz, CA, USA) at a concentration of 0.2 μg/ml as previously described [18]. The signal was detected and quantified using Scion Image v7.0 (Scion Corp., Frederick, MD, USA). All samples were normalized to actin signals. The results were used to categorize patients into three groups: high (TLR4/actin > 0.5), intermediate (0.15 ≤ TLR4/actin < 0.5), and low expressers (TLR4/actin < 0.15).

### Radiological features and grading of emphysema severity

Computed tomography (CT) images of all enrolled subjects were reviewed by an experienced chest radiologist (T.J.K.) and a pulmonologist (S.W.L.) without patients’ information. After jointly scoring 10 patients, who were not enrolled in this study, and adjusting their eye levels, the two readers independently reviewed the images. Any disagreements were discussed by the two readers, and another experienced pulmonologist (C.T.L.) helped in reaching an agreement. Preoperative CT images within 1 month before the resection were reviewed, and emphysema severity was scored by the Goddard classification [19]. Briefly, six CT images were selected for each patient: right and left fields of the upper lung (1 cm above the superior margin of the aortic arch), of the middle lung (1 cm below the carina), and of the lower lung (3 cm above top of the diaphragm). We scored each image from 0 to 3 according to the proportion of vascular disruption and the areas of low attenuation. A score of 0 meant no abnormality; 1, 0–25% involvement; 2, 25–50%; 3, 50–75%; and 4, 75–100% involvement. Emphysema was graded based on the total score of the six images: 0–7, mild; 8–15, moderate; and 16–18, severe emphysema.

CT scanning was performed during full inspiration using various CT scanners, including a Brilliance-64, MX-8000 IDT, and iCT 256 (Philips Medical Systems, Cleveland, OH, USA). Scanning was conducted from the thoracic inlet to the upper portion of the kidneys. Images were obtained using a window level of 600 Hounsfield units (HU), a window width of 1500 HU (lung window), a level of 30 HU, and a width of 400 HU (mediastinal window). Conventional CT images were obtained from the thoracic inlet to the lung base using a 3-mm section thickness.

### Pulmonary function test

All patients underwent a pulmonary function test within 1 month before resection. We used the results (without bronchodilation) that contained the largest forced expiratory volume in one second (FEV_1_). Spirometry was conducted by trained pulmonary technicians, according to the 2005 American Thoracic Society/European Respiratory Society recommendations [20] using a V_max_229 spirometer (Sensor-Medics, Yorba Linda, CA, USA).

### Exposure of rats to cigarette smoke

We performed animal experiments to exclude the possibility that smoking and acquired emphysema development themselves can affect TLR4 expression, therefore to make causal relationship more clearly. Three eight-week-old inbred female Lewis rats (Orient Bio, Seongnam, Korea) were exposed to smoke as previously described [21]. Three control animals inhaled only clean room air. The Institutional Animal Care and Use Committee of Asan Medical Center approved this study.

### Immunohistochemistry (IHC)

To analyze the major cells that resulted in a difference in TLR4 expression, immunohistochemical staining for TLR4 was performed in three randomly selected patients with emphysema who were low TLR4 expressers and three randomly selected patients without emphysema who were high TLR4 expressers. For the IHC analysis, 4-μm-thick sections were cut from the patient’s lung tissue blocks, deparaffinized in xylene, and rehydrated in a graded alcohol series. Antigen retrieval was performed in pH 6.0 citrate-phosphate buffer using a microwave oven for 15 min. The sections were incubated with anti-TLR4 antibody (1:50; Santa Cruz Biotechnology) for 1 h at room temperature. To detect the signals, an Envision kit (Dako, Glostrup, Denmark) was used according to the manufacturer’s instruction. An experienced pathologist (J.H.P.), who was blinded to the patient information, read the IHC findings. TLR4 expression was graded from 0 to 3 (0, no or very faint staining; 1, positive staining; 2, strong positive staining; 3, very strong positive staining).

### Statistical analysis

Emphysema severity and lung function according to TLR4 expression were compared using linear regression analysis. Comparison between groups was analyzed by Spearman correlation for continuous variables and the χ^2^ test for categorical variables. In the multivariate analysis, the backward selection method was used to exclude multi-collinearity. Statistical significance was assessed at a two-tailed *P*-value of 0.05. Agreements in the emphysema scores between the two readers were determined using the κ statistic [22]. All data are presented as means ± standard deviations. All statistical analyses were conducted using PASW software ver. 18.0 (SPSS, Inc., Chicago, IL, USA).

## Results

### Demographic characteristics

**Figure 1:**
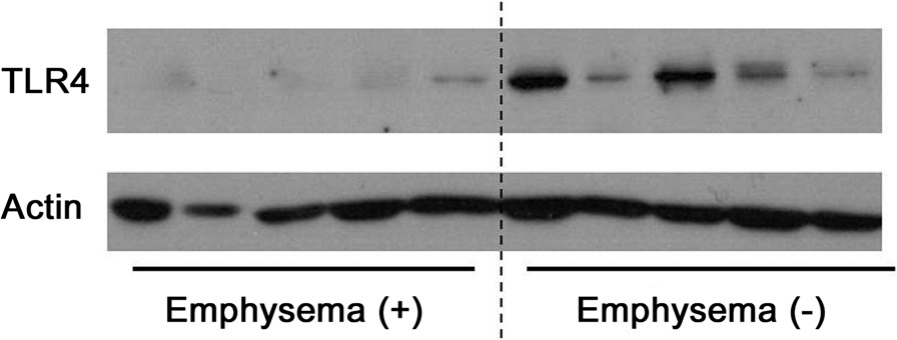
**The results of a pilot study.** Five smokers without emphysema showed higher TLR4 expressions than five smokers with emphysema. Each lane indicates TLR4 expression in lung of one patient.

Fifty-three patients were enrolled. The median age was 67 years (range, 37–78 years), and 51 patients (96.2%) were male. Mean smoking history was 31.3 ± 21.2 pack-years. Thirty nine patients (73.6%) were current smokers (smoking within 3 months before resection), and 11 (20.8%) patients had history of tuberculosis. Five patients (9.4%) were using inhaled corticosteroid before resection. A pilot study showed that TLR4 expression was lower in five patients with emphysema than in the other five without emphysema (Figure 1). A total of 33 (62.3%) patients were diagnosed with COPD by spirometry (FEV_1_/FVC < 0.7) [23].

### Emphysema severity and pulmonary function according to TLR4 expression

**Figure 2:**
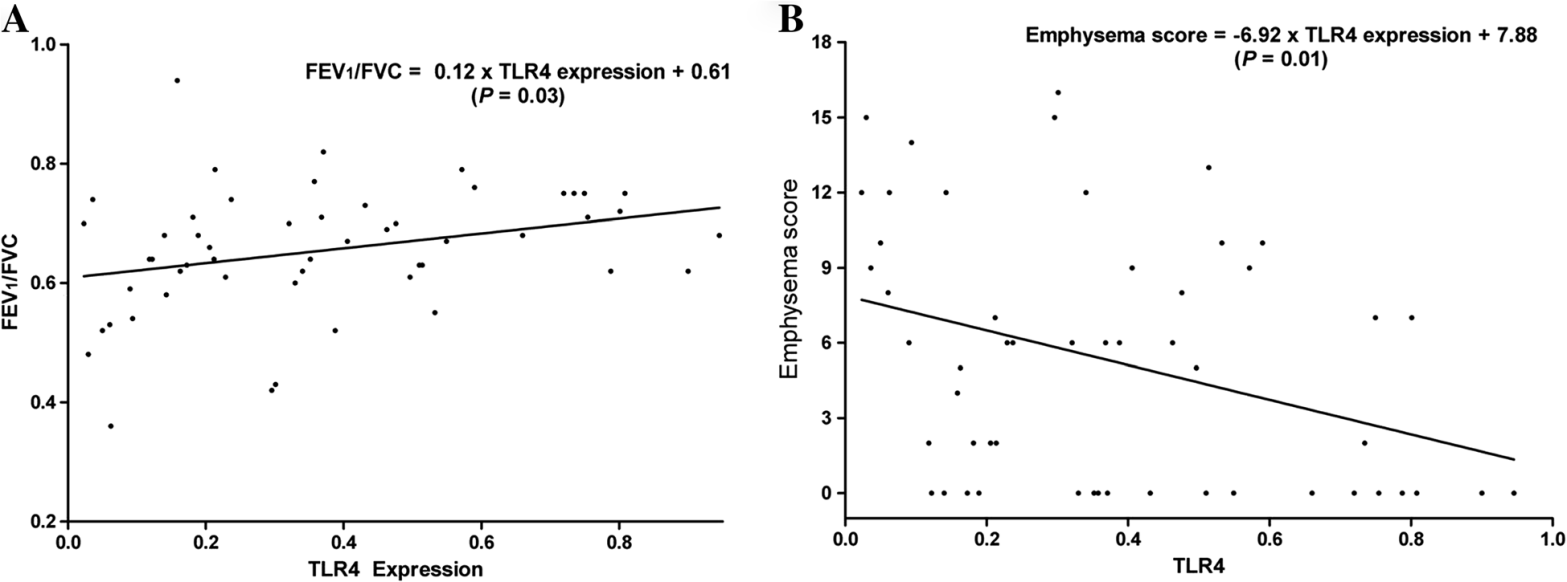
**Airflow limitation and emphysema severity according to TLR4 expression.** (**A**) FEV_1_/FVC increased and (**B**) emphysema score decreased as TLR4 expression increased. TLR4, Toll-like receptor 4; FEV_1_, Forced expiratory volume in one second; FVC, Forced vital capacity.

FEV_1_/FVC increased (*P*=0.03) and emphysema score decreased (*P*=0.01) as TLR4 expression increased (Figure 2). These were still significant, in multiple regression analysis including sex, age, tuberculosis history, smoking history and inhaled corticosteroid (ICS) usage within 3 months, which could affect pulmonary function and emphysema development [24-26]; *β* of TLR4 expression = 0.11 (95% confidence interval [CI] 0.007–0.22) for FEV_1_/FVC (*P* = 0.04) and −0.41 (95% CI = −13.1– -3.00) for emphysema score (*P* = 0.002).

**Figure 3:**
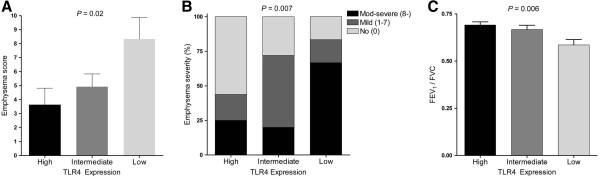
**Comparison of emphysema severity and pulmonary function between three groups according to TLR4 expression. **(**A**) Mean emphysema scores increased as TLR4 expression decreased. (**B**) Cases of severe emphysema were more common among patients with low TLR4 expression levels. (**C**) Airflow limitation was more severe at lower TLR4 expression levels. FEV_1_, Forced expiratory volume in one second; FVC, Forced vital capacity.

We categorized the patients into three groups according to TLR4 expression as described above. Twelve patients were low expressers, 25 patients were intermediate expressers, and 16 patients were high expressers. No differences in age, gender, tuberculosis, or smoking history were observed among the groups (Table 1). The mean emphysema score increased as TLR4 expression decreased; the mean emphysema scores of the high, intermediate, and low expressers were 3.63 ± 4.77, 4.92 ± 4.60, and 8.33 ± 5.26, respectively (*P* = 0.02, Figure 3A). The TLR4 low expresser group included a higher number of severe-stage emphysema cases; the proportions of severe compared with mild emphysema in the high, intermediate, and low expressers were 43.8% (7/16), 72.0% (18/25), and 83.4% (10/12) (*P* = 0.007, Figure 3B). Airflow limitation also became severer with lower levels of TLR4 expression; the mean FEV_1_ per forced vital capacity (FVC) values in the high, intermediate, and low expressers were 0.69 ± 0.07, 0.67 ± 0.11, and 0.58 ± 0.11 (*P* = 0.006, Figure 3C). However, there was no significant difference in patients with COPD (8/16 [50.0%] in high, 15/25 [60.0%] in intermediate and 10/12 [82.3%] in low expressers, respectively, *P*=0.19) and FEV_1_ per its predicted value (90.8 ± 18.0 in high, 93.2 ± 17.3 and 86.2 ± 11.0 in low expressers, respectively, *P*=0.52) among groups. TLR4 expression was higher in patients without COPD than with COPD (0.45 ± 0.26 and 0.33 ± 0.25), but it was not statistically significant (*P*=0.11).

**Table 1:** Comparison of characteristics and pulmonary function between the groups

**Characteristic**	**Low Expressers**	**Intermediate Expressers**	**High Expressers**	***P*-value**
	**(TLR4/actin < 0.15)**	**(0.15 ≤ TLR4/actin < 0.5)**	**(TLR4/actin ≥ 0.5)**	
	**N = 12**	**N = 25**	**N = 16**	
Age, years	65.3 ± 8.3	64.0 ± 9.2	67.4 ± 7.8	0.49
Male gender, n (%)	12 (100)	24 (96.0)	15 (93.8)	0.69
Smoking, pack-years	38.8 ± 27.2	35.6 ± 17.8	38.8 ± 22.3	0.87
Current smoker, n (%)	10 (83.3)	20 (80.0)	9 (56.3)	0.17
TB history present, n (%)	2 (16.7)	6 (24.0)	3 (18.7)	0.85
ICS users^*^, n (%)	2 (16.7)	2 (8.0)	1 (6.3)	0.61

TLR4, toll-like receptor 4; TB, tuberculosis; ICS, inhaled corticosteroid; FEV_1_, forced expiratory volume in one second; FVC, forced vital capacity; COPD, chronic obstructive pulmonary disease.

*The number of patients with inhaled corticosteroid prescribed within 3 months of lung resection. Fluticasone 250mcg with formoterol 50mcg twice a day was prescribed in four patients and fluticasone 500mcg with formoterol 50mcg twice a day in one low expresser.

Agreement between the two readers (T.J.K. and S.W.L.) for emphysema grades was almost perfect (κ = 0.82, *P* < 0.001). The differences in emphysema scores between the two readers were 0 for 30 (56.6%) patients, 1 for 12 (22.6%) patients, 2 for nine (17.0%) patients, and ≥3 for two (3.8%) patients.

### Major cells resulting in TLR4 expression differences in lung tissue

**Figure 4:**
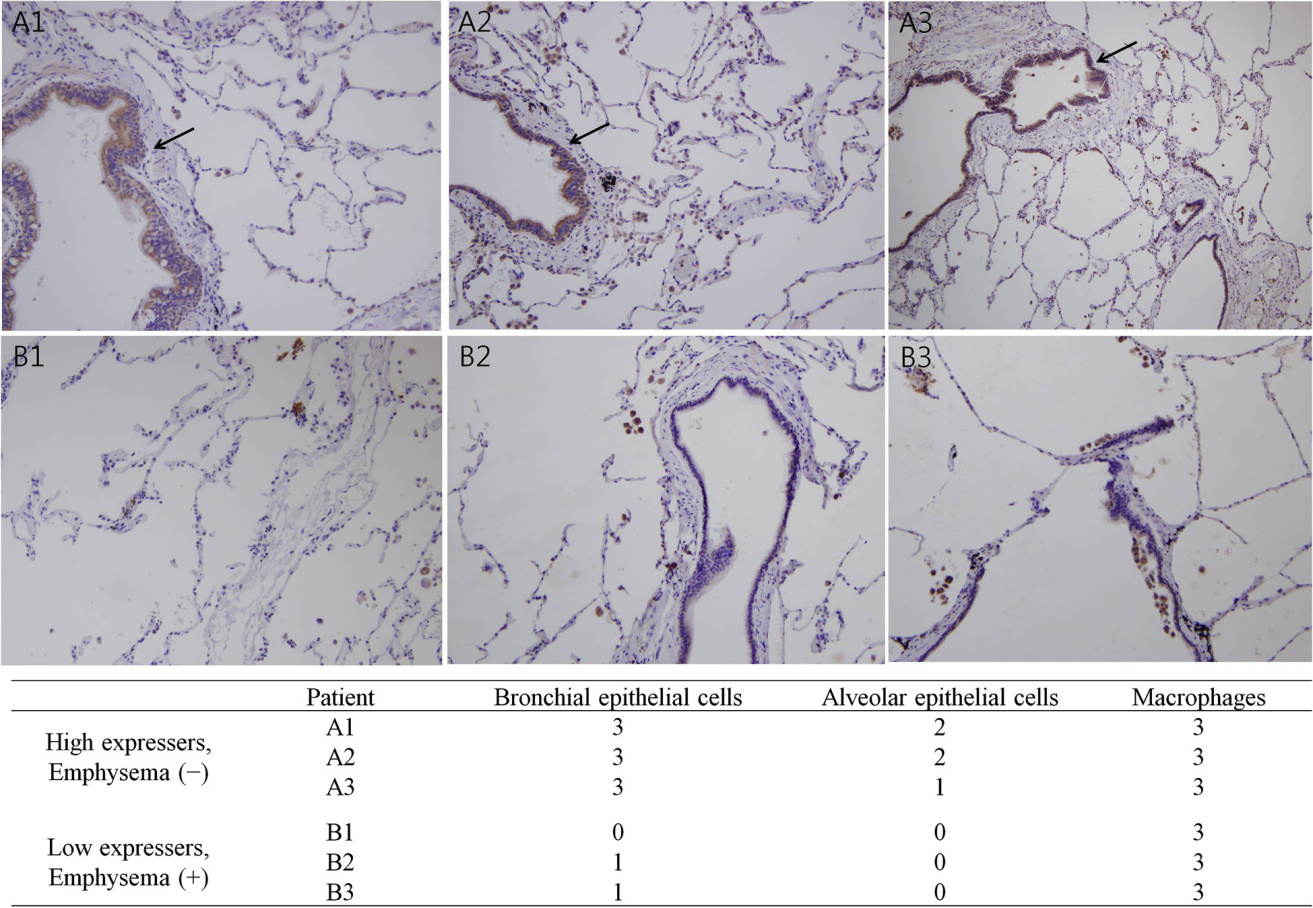
**Toll-like receptor 4 (TLR4) immunohistochemistry of lung tissues patients. **(**A**) Tissues from high TLR4 expressers without emphysema. Arrows indicate high TLR4 expressions in bronchial epithelium. (**B**) Tissues from low TLR4 expressers with emphysema. Immunohistochemical staining shows that bronchial and alveolar epithelial cells exhibited major differences in TLR4 expression (×200). An experienced pathologist (J.H.P.), who was blinded to the patient information, read the IHC findings. TLR4 expression was graded from 0 to 3 (0, no or very faint staining; 1, positive staining; 2, strong positive staining; 3, very strong positive staining).

Immunohistochemical staining for TLR4 showed that bronchial and alveolar epithelial cells were the major cells exhibiting differences in TLR4 expression. Alveolar macrophages showed high TLR4 expression in all patients and thus did not contribute to the difference in expression among groups (Figure 4).

### TLR4 Expression among inbred rats according to acquired emphysema development

**Figure 5:**
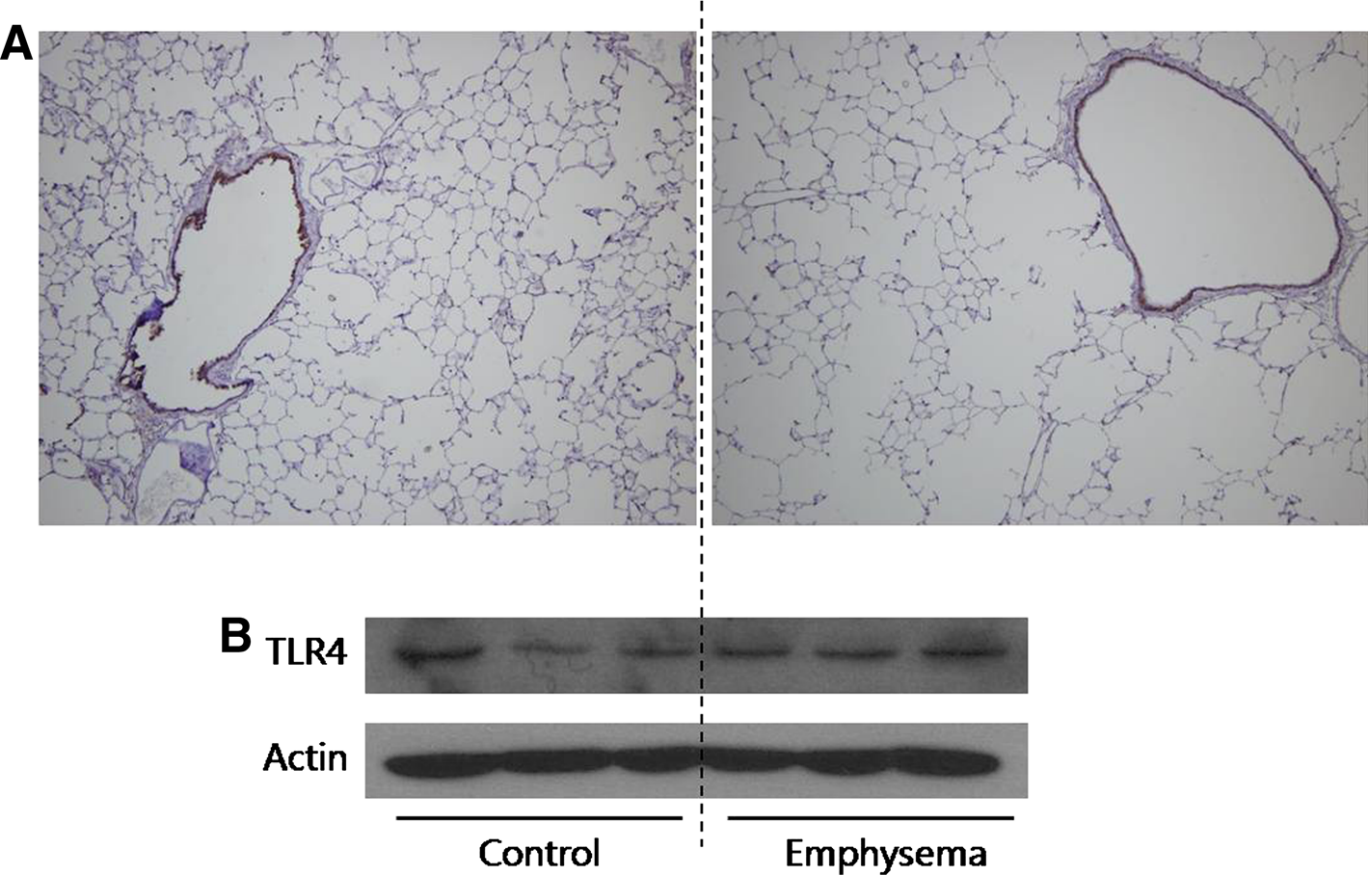
**Toll-like receptor 4 (TLR4) expressions in lung tissues of inbred rats with and without emphysema. **(**A**) Lung tissue shows the development of emphysema after 6 months of exposure to smoke. However, The IHC stain of TLR4 showed no difference in expression between rats with and without emphysema (×40). (**B**) TLR4 expression in the lungs is not different between smoke-exposed and control rats in Western blot analysis.

We compared TLR4 expression between inbred rats with and without emphysema to exclude the possibility that acquired emphysema development itself caused by smoking can affect TLR4 expression. We confirmed emphysema development after 6 months of smoke exposure. However, no difference in TLR4 expression was observed between rats with and without emphysema (Figure 5).

## Discussion

We showed that a down-regulated TLR4 expression in the lung can be a predictor or biomarker of emphysema in smokers or biomarker of emphysema, as patients with down-regulated TLR4 expression had a greater probability of developing emphysema and airflow limitation. Differences in TLR4 expression were attributable mainly to expression levels in bronchial and alveolar epithelial cells. Smoke-induced emphysema itself did not appear to alter TLR4 expression; therefore TLR4 expression seems to affect emphysema development or progression, rather than vice versa.

Several studies have shown that defects in innate TLR4-mediated immunity can be associated with emphysema. TLR4 knockout causes spontaneous emphysema in animals [17]. The presence of the TLR4-T339I polymorphism is associated with a 2.4-fold increased risk for developing COPD, highlighting the relationship between impaired innate immunity and COPD development [27]. The Gly299 allele is present at a decreased frequency among patients with COPD and may be absent from COPD patients who have never smoked [28]. Our findings can make an important contribution on the relationship between innate immunity and COPD, because they show that down-regulated TLR4 expression in the lung may be related to emphysema in humans, as in animals.

An association between TLR4 expression and COPD has also been noted previously. Most studies have suggested that TLR4 expression is decreased in the blood of patients with COPD. The peripheral blood T_H_1 cell response to lipopolysaccharide (LPS) is impaired in patients with COPD compared with the response in never-smokers, and TLR4 overexpression via transfection restores this impairment [29]. Compared with less severe disease, severe COPD is associated with reduced TLR4 expression in the nasal epithelium [24]. In contrast to some positive results in blood, cells in a sputum analysis failed to show this relationship. TLR4 expression on sputum neutrophils was not different in COPD patients [30], and TLR4 mRNA in induced sputum did not differ significantly between COPD patients and healthy controls [31]. A previous study showed no significant difference in TLR4 expression in epithelial cells between COPD and normal subjects, but several factors such as smoking history, age and sex were not controlled and small number subjects limited the power of test [32]. This study is meaningful because we compare the TLR4 expression among smokers. Previous studies compare the expression between smoker COPD and never-smoker controls [25,33].

Theoretically, TLR4 deficiency may partially protect against smoke-induced emphysema, because TLR4 deficiency partially prevents smoke-induced influx of dendritic cells, lymphocytes, and neutrophils [16]. Clear correlations have been found between the numbers of these inflammatory cells and the severity of COPD [34,35]. However, previous studies and our study have shown that TLR4 deficiency does not protect against the development of emphysema. To the contrary, it appears to be related with emphysema development, which can be explained by the role of TLR4 in the respiratory system. In TLR4 deficient mouse, NADPH oxidase was up-regulated in lungs, resulting in increased oxidant generation and elastolytic activity [17]. Expression of the autophagic protein LC3B and markers of cell death in response to cigarette smoke exposure were increased in epithelial cells from TLR4 deficient mice [33]. Overall, TLR4 exerts a protective role with respect to smoke-induced emphysema development. It is not still evident whether the same mechanism is applied to human and further study is necessary. TLR4 expression seemed associated with emphysema severity which was reported to be more correlated with airflow limitation (FEV_1_/FVC) than COPD stage defined by FEV_1_ (% predicted) [36].

Several TLR4-dependent mechanisms are likely to be involved in cigarette smoke-induced pulmonary inflammation. Smoke may activate TLR4 signaling on pulmonary epithelial cells and transmigrated resident cells such as macrophages, which act as the first line of defense against external threats [16,37]. Innate immunity mediated by TLR4 also triggers the first-line host defense response to Gram-negative bacterial infections and is crucial for initiating subsequent T cell-mediated adaptive immune responses [37]. TLR4 expression on the respiratory epithelium allows for rapid activation of host defenses against outside stimuli such as smoke and bacteria, resulting in the induction of inflammatory mediators and antimicrobial peptides. The increased risk for bacterial infection in patients with COPD may be caused by an inability to effectively clear bacteria and by misguided inflammatory responses. Emphysema develops due to chronic inflammation and impaired matrix and cellular repair. Thus, an impaired defense owing to TLR4 deficiency combined with repeated inflammation may result in the development of emphysema. However, it is unclear whether the reduced TLR4 expression in patients with COPD is an adaptive response to increased exposure to external threats such as Gram-negative bacteria or smoke, as part of the phenomenon of endotoxin tolerance.

Several limitations of this study should be noted. First, the causal relationship between down-regulated TLR4 expression and emphysema is ambiguous. To make a firm conclusion, smokers should be followed for several decades after obtaining lung tissues or respiratory cells; however, such studies are not practical and present ethical issues. Similar experiment using other tissues or blood-born cells could have clarified this issue more, but we could not because they were not available. However, our data showed that smoke-induced emphysema itself did not affect TLR4 expression, providing more evidence that down-regulated TLR4 expression results in emphysema. Previous reports showing that TLR4 knockdown induces spontaneous emphysema also support this idea [17]. Second, TLR4 expression can be induced or decreased by LPS stimulation [29,38]. Thus, TLR4 expression levels can vary with different clinical conditions such as Gram-negative bacterial infections. In our study, lung tissues were taken during elective lung resection, when patients were clinically stable without evidence of pulmonary infection or exacerbation. Third, we could not find a causable gene that could explain the differential TLR4 expression. Although we examined several single-nucleotide polymorphisms in the *TLR4* gene, including minor alleles with frequencies >5% in the HAP map of Asians, no significant results were identified (data not shown). Epigenetic factors may be associated with the differential expression of TLR4. Fourth, functional aspects by down-regulation of TLR4 expression, such as cytokine secretion, could not be evaluated and because of the retrospective nature of this study.

## Conclusion

In conclusion, down-regulated TLR4 expression in lung was associated with emphysema and airflow limitation in smokers. This report will broaden our understanding of COPD pathogenesis, although further studies are required to clarify a causal relationship between down-regulated TLR4 expression and emphysema.

## Competing interests

The authors declare that they have no competing interests.

## Authors’ contribution

SWL and CTL conceived the idea. SWL, DRK and JWH performed experiments. SWL, TJK, and CTL interpreted the radiologic findings. JHP and JHJ interpreted the pathologic findings. SHJ provided important reagent. SWL, JHL, and CTL analyzed the data and drafted the manuscript. All authors read and approved the final manuscript.
